# Influencing factors of suicide in hospitalized patients with mental disorders: a retrospective study using medical malpractice cases in China

**DOI:** 10.3389/fpsyt.2025.1545173

**Published:** 2025-07-21

**Authors:** Qiang Wang, Daming Sun, Guangjian Li, Lin Li, Hongyan Li, Yajun Xu

**Affiliations:** ^1^ Law School of Nanjing University, Nanjing University, Nanjing, China; ^2^ Forensic Science Center, East China University of Political Science and Law, Shanghai, China; ^3^ Department of Psychology, Wannan Medical College, Wuhu, China; ^4^ School of Law, Inner Mongolia University, Hohhot, China; ^5^ School of Basic Medicine, Nanjing Medical University, Nanjing, China

**Keywords:** suicide, mental disorders, hospitalized patients, medical malpractice, influencing factors, forensic psychiatry

## Abstract

**Objective:**

This study aims to reveal the suicide risk factors in specific situations of medical malpractice.

**Methods:**

We conducted a systematic analysis using the medical malpractice case data from the judgment search system on China Judgments Online, covering the years 2012 to 2022. The χ^2^ test was applied to compare group differences, while a binary logistics regression analysis was used to assess factors influencing suicide among hospitalized patients with mental disorders.

**Results:**

A total of 268 finalized medical malpractice judgments were analyzed. From 2012 to 2022, medical malpractice cases involving psychiatric inpatients in China show a significant upward trend. Suicide cases of hospitalized patients with mental disorders accounted for 32.5% of all medical malpractice cases. These incidents were more frequent in comprehensive hospitals, tertiary hospitals, and economically developed regions such as East China and Central China. Chi-square testing and multivariate binary logistic regression analysis revealed that hospital type, history of suicidal behavior, clinical psychiatric diagnosis of others, medical fault in violating specific regulations, and medical fault in breach of duty of care were identified as independent risk factors (OR=2.662,3.866,3.567,3.247,3.593; 95% CI: 1.368-5.180, 1.473-10.146, 0.398-9.107,1.193-8.838,1.406-9.179).

**Conclusions:**

In China, the proportion of suicide cases of hospitalized psychiatric patients in civil cases of medical malpractices has been increasing. Independent risk factors include hospital type, suicidal history, history of suicidal behavior, clinical psychiatric diagnosis of others, medical fault in violating specific regulations and medical fault in breach of duty of care. Optimizing resource allocation in comprehensive hospitals, enhancing ward safety, and standardizing clinical protocols are urgently needed to mitigate preventable suicides in this vulnerable population.

## 1 Introduction

Deaths among hospitalized patients with mental disorders can be classified as either natural or unnatural. Research shows that psychiatric populations experience higher “all-cause” mortality rates than the general public or other patient groups, with a significant portion of these deaths resulting from “unnatural causes” like accidents or suicides (1). Numerous factors contribute to unnatural deaths. Hospitalized patients with mental disorders often face an increased risk of suicide, particularly during the acute phase (2, 3). Despite medical institutions conducting suicide risk assessments upon admission and developing treatment plans accordingly, suicide remains a significant cause of death among patients with severe mental disorders (4). Additionally, factors such as the adequacy of ward safety measures, potential negligence by nursing staff (nurses, doctors, and other clinicians), and compliance with established norms in the use of compulsory measures during patient diagnosis and treatment can also contribute to patient fatalities (5–9).

It is well established that patients seeking hospital treatment for mental disorders face a heightened risk of suicide (10). A study of 648,646 patients visiting emergency departments in California revealed that, compared to the general population, those who engage in deliberate self-harm have a suicide mortality rate 56.8 times greater, those with suicidal ideation have a rate 31.4 times higher, and individuals with any other primary issues have a rate 1.9 times higher. Additionally, there is an increased risk of other causes of death (11). For individuals admitted with mental disorders, it is imperative to devise effective strategies to prevent suicide (12).

A number of studies have attempted to predict the factors that contribute to the suicide of inpatients with mental disorders. However, little has been written about the post-event factors affecting the suicide of inpatients with mental illnesses. According to China’s medical malpractice judicial appraisal industry standard, the “Guidance for Judicial Expertise of Medical Malpractice (China SF/T 0097–2021),” medical malpractice is defined as the harm caused to patients by errors made by medical institutions or personnel during diagnosis and treatment. This definition aligns closely with those in other countries (13). Following incidents of medical malpractice, patients and their legal representatives often claim medical institutions to bear corresponding legal liabilities through litigation or mediation procedures (14). Among malpractice claims against psychiatrists, completed suicides are the most common grounds for seeking compensation, as they are the primary catalyst for initiating malpractice lawsuits (15). A psychiatrist may face a 1 in 3 likelihood of encountering a malpractice claim if an inpatient under their care dies by suicide during hospitalization (16). Suicide among hospitalized persons with mental disorders places significant financial, emotional, and moral pressures on health care providers and institutions (17). Thus, conducting a systematic analysis of judicial documents from medical malpractice cases offers insights into the influencing factors of suicide in patients with mental disorders during hospitalization (18, 19).

In this context, we examined 268 cases involving hospitalized patients with mental disorders. The main objective of this study was to explore the factors that influence suicide in hospitalized patients with mental disorders based on the context of the occurrence of medical errors and to provide guidance.

## 2 Materials and methods

### 2.1 Data sources

We conducted a thorough search of the judgment search system database on China Judgments Online, maintained by the Supreme People’s Court of the People’s Republic of China (https://wenshu.court.gov.cn/). China Judgments Online is a unified national platform for court judgment disclosure established in 2013 by the Supreme People’s Court of the People’s Republic of China. Along with the trial process disclosure platform and the enforcement information disclosure platform, it is one of the three major judicial openness platforms. The platform encompasses all publicly accessible judicial documents pertaining to civil cases, criminal cases, and administrative cases. In this study, the keywords “mental disorder” and “medical malpractice” were used to cover cases dated between January 1, 2012, and December 31, 2022, and limited to civil cases of the first instance. A total of 789 judgment documents were found to be relevant to this search. To ensure alignment with the research objectives, exclusion criteria were applied to the dataset. Exclusions include: 1) cases with no medical harm occurring during hospitalization; 2) incidents arising from outpatient visits or consultations; 3) cases with unclear or ambiguous mental disorder diagnoses; and 4) cases unrelated to psychiatric hospitals or psychiatric departments within comprehensive hospitals. Based on these criteria, 268 cases formed the core dataset for this study.

### 2.2 Data collection tools

#### 2.2.1 Guidance for judicial expertise of medical malpractice

The *Guidance for Judicial Expertise of Medical Malpractice* (China SF/T 0097–2021), developed in 2021 by the Academy of Forensic Science along with several domestic appraisal institutions, addresses the requirements of people’s courts in civil litigation trials and the unique demands of public legal services outside litigation. It provides procedures for identification, evaluation of damage consequences, and analysis of causality, fully meeting judicial needs (20). The guidance categorizes the outcomes of medical malpractice into classifications such as death, disability, and extended disease duration. Furthermore, it classifies medical errors into specific categories: violations of explicit regulations (where medical institutions or staff breach laws, administrative rules, or specific diagnostic, treatment, and nursing standards), breaches of duty of care (where medical institutions or personnel, despite being capable of preventing harm, fail to do so due to negligence or overconfidence), and failures in fulfilling the duty to inform (where institutions or staff neglect to inform patients of their condition or proposed medical interventions and do not secure informed consent).

#### 2.2.2 Self-designed scale

A self-designed scale was employed to assess medical malpractice cases’ general characteristics. Key aspects included hospital type, level, region, and clinical diagnosis. The hospital type was comprehensive or psychiatric. Psychiatric hospitals refer to those treating mental illnesses, such as mental health centers. Comprehensive hospitals are defined as a facility covering a wide range of diseases, equipped with departments such as Psychiatry, Internal Medicine, Surgery, Obstetrics and Gynecology, and Pediatrics. Patients often choose comprehensive hospitals due to mental illness stigma. The patients involved in this study are those who experienced medical harm during outpatient or inpatient treatment in the psychiatric departments of a comprehensive hospital. In China, hospitals are classified into three tiers: primary, secondary, and tertiary, based on their functions, facilities, technical capabilities, and management. Each tier is subdivided into grades A, B, and C, with tertiary hospitals also including a Special Grade. Specifically, tertiary hospitals possess robust medical technologies and scientific research capacities, typically functioning as large comprehensive hospitals. This tier-based classification system is uniformly implemented nationwide and remains unaffected by hospital operational background or ownership structure. In our study, we categorized medical facilities into First-class and below, Second-class, and Tertiary hospitals determined by the hospital grade. Provinces were categorized according to China’s geographical and climatic regions: East China, Central China, North and Northeast China, South China, and Northwest and Southwest regions (https://zhidao.baidu.com/question/196923419.html).

Clinical psychiatric diagnoses were defined as previous clinical diagnoses recorded in medical records, including outpatient and discharge diagnoses. Among them, patients with mental disorders admitted to the general hospital for treatment included those who were not diagnosed with mental disorders at the first outpatient clinic. However, they were confirmed as mental disorders after comprehensive consultation and admitted to the general hospital for treatment. Variables such as a history of suicidal behavior, suicide risk assessment, medical fault in breach of duty to inform (the possible social risks of hospitalization and non-hospitalization, the purpose, methods, benefits and risks of the proposed treatment measures, and the risks and benefits of refusing such measures, etc.), medical fault in violation of specific regulations (diagnosis errors, medication errors, special treatment errors, etc.) and medical fault in breach of duty of care (failure to fulfill security obligations; Causing damage to patients or others due to negligent management or safety problems of hospital equipment; The patient’s condition was delayed due to negligence; etc.) were recorded as binary responses (“Yes” or “No”).

To ensure personal information protection, court documents do not include patients’ general demographic data such as name, gender, age, or educational background. Our analysis focuses exclusively on: 1) basic institutional details of medical facilities (e.g., hospital grade and category, supplemented by accessing official websites for verification); 2) diagnoses of hospitalized individuals with mental disorders; and 3) contextual information related to suicide incidents, including time, location, and method. This approach prioritizes privacy compliance while retaining essential data for research and legal purposes.

### 2.3 Data analysis

A retrospective descriptive analysis of the sample was first conducted. Count data were expressed as absolute numbers or percentages. We then divided the sample into suicide and non-suicide groups, and chi-square test was used for comparison between groups. In the final phase, we conducted binary logistic regression analysis with the presence of suicide among hospitalized psychiatric patients as the dependent variable, using variables that showed statistical significance in the univariate analysis as independent variables. All analyses were conducted using the Statistical Package for Social Sciences (SPSS) version 26.0, with *p* < 0.05 considered indicative of statistical significance.

## 3 Results

### 3.1 Analysis of time trend and characteristics of medical malpractice cases

During the period 2012-2022, 789 cases of medical malpractice were identified, with 268 (34.0%) meeting the inclusion criteria; 32.5% of hospitalized mentally ill patients committed suicide, and 77.24% of the cases involved medical institutions making medical errors. Among these 268 cases of hospitalized patients with mental disorders, **Table 1** presents the most important characteristics. The main characteristics identified in these cases are as follows: 1) treatment in comprehensive hospitals; 2) predominance in tertiary hospitals; 3) a high incidence in Eastern China; 4) a low incidence of suicidal behavior among hospitalized patients with mental disorders; 5) a low frequency of suicide risk assessments conducted post-hospitalization; 6) a relatively high proportion of patients diagnosed with schizophrenia, schizotypal, and delusional disorders; and 7) violations in breach of duty of care as the primary type of medical malpractice observed.

**Table 1:** Characteristics of 268 cases of psychiatric medical malpractice.

Variable		Suicidal (%)	Non-suicidal (%)	Total number	χ2	P-values
Type of hospital	Comprehensive hospitals	63 (41.2)	90 (58.8)	153	12.348	0.000
	Specialized psychiatric hospitals	24 (20.9)	91 (79.1)	115		
Hospital level	First and below class hospital	23 (41.8)	32 (58.2)	55	2.811	0.245
	Second-class hospital	26 (29.2)	63 (70.8)	89		
	Tertiary hospital	38 (30.6)	86 (69.4)	124		
Province	Northeast China and North China	12 (21.1)	45 (78.9)	57	6.408	0.093
	Central China	19 (36.8)	38 (63.2)	57		
	Eastern China	36 (40.9)	52 (59.1)	88		
	South China, northwest and southwest China	20 (30.3)	46 (69.7)	66		
History of suicidal behavior	Yes	25 (71.4)	10 (28.6)	35	27.880	0.000
	No	62 (26.6)	171 (73.4)	233		
Assessment of suicide risk	Yes	35 (46.7)	40 (53.3)	75	9.584	0.002
	No	52 (26.9)	141 (73.1)	193		
Clinical psychiatric diagnosis (ICD-10)	Schizophrenia, schizotypal and delusional disorders	37 (29.4)	89 (70.6)	126	38.371	0.000
	Mood disorders	41 (55.4)	33 (44.6)	74		
	Others	9 (13.2)	59 (86.8)	68		
Medical fault in breach of duty to inform	Yes	6 (27.3)	16 (72.7)	22	0.294	0.587
	No	81 (32.9)	165 (67.1)	246		
Medical fault in violating specific regulations	Yes	72 (48.3)	77 (51.7)	149	38.497	0.000
	No	15 (12.6)	104 (87.4)	119		
Medical fault in breach of duty of care	Yes	71 (52.2)	65 (47.8)	132	49.092	0.000
	No	16 (12.1)	16 (87.9)	136		

**Figure 1:**
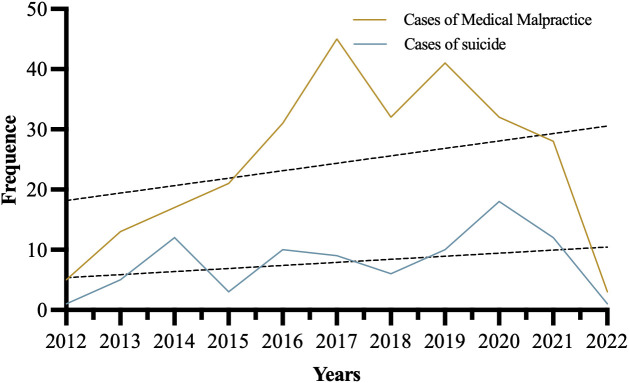
Temporal variation trend of medical malpractice data of hospitalized patients with mental disorders.

As shown in **Figure 1**, malpractice and suicide cases among patients with mental disorders are distributed annually. According to the findings, the number of such cases has been increasing over time. It is worth noting that China Judgments Online’s relatively slow uploading of court decisions may explain the relatively low number of medical injury judicial authentication cases reported in 2022. Additionally, since 2020, medical malpractice cases and suicide cases involving hospitalized psychiatric patients have shown a declining trend. However, this result is not necessarily objective or accurate due to the impact of the COVID-19 pandemic. Judicial operations could not proceed smoothly, and even if medical malpractice or suicides occurred among these patients, the processing speed of such cases slowed significantly. This delay affected the conclusion time of adjudications, thereby leading to a portion of judgments not being published online.

### 3.2 Comparison of the characteristics of medical malpractice cases between suicidal and non-suicidal patients

The incidence of medical malpractice cases in comprehensive hospitals (57.1%, n=153) was significantly higher than in psychiatric hospitals (42.9%, n=115), with a statistically significant difference observed [χ^2^ = 12.348, *p* < 0.01]. By hospital level, tertiary hospitals accounted for a higher number of cases (46.2%, n=124), though the difference in malpractice case frequency across hospital levels did not reach statistical significance, the proportion for tertiary hospitals remained high [χ^2^ = 2.811, *p* = 0.245]. Regionally, East China exhibited a higher incidence of medical malpractice cases (32.9%, n=88), although differences across regions were not statistically significant [χ^2^ = 6.408, *p* = 0.093]. Please refer to **Table 1** for detailed specifics.

Significant differences were observed between suicidal and non-suicidal patients in terms of history of suicidal behavior, suicide risk assessment and clinical psychiatric diagnosis among hospitalized patients with mental disorders [χ2 = 27.880, 9.584, 38.371, p < 0.01]. Patients with schizophrenia, schizotypal, and delusional disorders represented a high-risk group in medical malpractice cases involving hospitalized patients with mental disorders (47.0%, n=126). However, effective judgments contained limited records on whether psychiatric patients exhibited suicidal behavior upon admission (86.9%, n=233) or whether hospitals conducted suicide risk assessments (72.0%, n=193). Please refer to **Table 1** for the detailed specifics.

By further comparison of the characteristics of medical errors in medical institutions, there were statistically significant differences in violating specific regulations and violations of duty of care between suicidal and non-suicidal patients [χ^2^ = 38.497, 49.092, *p* < 0.01]. However, no significant difference was found for medical fault related to breaches of duty to inform [χ^2^ = 0.294, *p* = 0.587]. Please refer to **Table 1** for the detailed specifics.

### 3.3 Characteristics of in-hospital suicide among patients with mental disorders

In the death cases of hospitalized patients with mental disorders, the causes of death could be divided into suicide (47.5%, n=87), sudden death (14.8%, n=27), self-disease (24.6%, n=45), craniocerebral trauma (3.8%, n=7), accident (5.5%, n=10) and unknown cause (3.8%, n=7). An analysis of 87 cases of suicide among hospitalized individuals with mental disorders revealed the following characteristics: 1) a notable increase in suicides occurring on weekend night; 2) a heightened risk of suicide within the first week of admission; 3) a higher incidence of suicides taking place within the ward; 4) hanging and jumping from heights are the predominant methods of suicide; 5) most patients utilized bed sheets or clothes to commit suicide; and 6) windows were the preferred point of egress for those who chose to jump. Please refer to **Table 2** for more information.

**Table 2:** Characteristics of 87 in-hospital suicide among patients with mental disorders.

Variable		N (%)
Suicide time	Weekday daytime	15 (17.3)
	Weekday night	4 (4.6)
	Weekend daytime	25 (28.7)
	Weekend night	39 (44.8)
	Null	4 (4.6)
Suicide period	1-7days	42 (48.3)
	8-31days	20 (23.0)
	31days-	18 (20.7)
	Null	7 (8.0)
Suicide location	Bedroom	31 (35.6)
	Bathroom	33 (37.9)
	Suicide out of hospital	19 (21.8)
	Null	4 (4.61)
Methods of suicide	hanging	37 (42.6)
	jumping from a height	31 (35.6)
	Null	19 (21.8)
Rope of hanging	Bed sheet	4 (10.8)
	Clothes	6 (16.2)
	shoelace	3 (8.1)
	Shower	1 (2.7)
	Television cable	1 (2.7)
	towel	2 (5.4)
	Null	20 (54.1)
Location of jumping	Windows	18 (58.1)
	staircase	3 (9.6)
	roof	4 (12.9)
	Null	6 (19.4)

Suicide out of hospital:Suicide out of hospital refers to the situation where a person with mental disorders who has been admitted to the hospital commits suicide outside the hospital after escaping.

### 3.4 Analysis of the factors influencing the suicide of psychiatric inpatients

A multivariate binary logistic regression analysis was conducted to identify factors predicting patient suicide. The analysis included variables that were statistically significant in univariate analysis, using a forward likelihood ratio (LR) method for model construction. The Hosmer-Lemeshow test confirmed the model’s good fit (χ² = 6.311, P = 0.504). The results showed that hospital type, history of suicidal behavior, clinical psychiatric diagnosis of others, medical fault in violating specific regulations, and medical fault in breach of duty of care were independent predictors of patient suicide (P < 0.05). Among these, hospital type, history of suicidal behavior, clinical psychiatric diagnosis of others, medical fault in violating specific regulations and medical fault in breach of duty of care were identified as risk factors (OR > 1), as shown in **Table 3**.

**Table 3:** Influencing factors of suicide in hospitalized patients with mental disorders.

Variable		OR	P value	95%CI
Type of hospital	Comprehensive hospitals	2.662	0.004	1.368-5.180
	Specialized psychiatric hospitals	1		
History of suicidal behavior	Yes	3.866	0.006	1.473-10.146
	No	1		
Assessment of suicide risk	Yes	0.661	0.285	0.309-1.413
	No	1		
Clinical psychiatric diagnosis (ICD-10)	Mood disorders	1.551	0.228	0.760-3.168
	Others	3.567	0.008	0.398-9.107
	Schizophrenia or other primary psychotic disorders	1		
Medical fault in violating specific regulations	Yes	3.247	0.021	1.193-8.838
	No	1		
Medical fault in breach of duty of care	Yes	3.593	0.008	1.406-9.179
	No	1		

Cox & Snell R Square=0.291; Nagelkerke R Square=0.407; OR, Odds ratio; 95%CI, 95% confidence interval.

## 4 Discussion

### 4.1 Predominant profiles of suicides among inpatients with mental disorders

This study examined the characteristics of psychiatric medical malpractice in China through a retrospective analysis of legally binding court rulings in related cases. It focused on factors influencing suicide among hospitalized individuals with mental disorders. Our findings indicated a steady increase in medical malpractice cases involving psychiatric inpatients from 2012 to 2022. Similarly, suicide rates among hospitalized patients also increased. This observation is consistent with results from other studies (21), highlighting ongoing challenges in adequately protecting psychiatric inpatients’ health and life rights. It should be noted that because the sample of this study came from valid judgments, it cannot accurately reflect the suicide rate of people with mental disorders admitted to the hospital.

Analysis of inpatient suicide among psychiatric patients demonstrates a heightened suicide risk during the initial hospitalization week, particularly pronounced in male populations, aligning with previous study findings (22). The high likelihood of suicide among patients with mental disorders during the first week of hospitalization may be due to them typically being in the acute phase of their illness upon admission. Moreover, our research has also revealed a high probability of suicide among patients admitted to mental disorders during the weekend nights. On the one hand, nocturnal wakefulness poses a significant risk factor for suicide and suicidal ideation in clinical populations (22, 23). Inpatients with mental disorders and insomnia should be given special attention by their physicians. On the other hand, a reduction in the number of doctors on duty on weekends and night shifts is known to lead to lapses in attention. This results in a noticeably lower quality of patient supervision than during normal working hours.

Our research found that patients commit suicide using clothing and bedsheets. While patients are typically required to undergo an inventory check upon admission, essential items such as bed sheets and clothing are exempted from this scrutiny. Given that confiscating such personal items is impractical, medical institutions should acknowledge the potential for these necessities being utilized as a means of suicide. To mitigate this risk, healthcare providers could consider supplying patients with specialized materials for their bedding and attire, such as garments made from soft, weakly tension-resistant fabrics, which cannot support significant weight, thereby reducing the likelihood of their use in suicide attempts. Furthermore, medical institutions might explore upgrading their ward facilities with advanced smart devices. For instance, the intelligent psychiatric hospital management platform offered by MadoCloud Medical Technology Co., Ltd., features modules for smart supervision, nursing, and early warning. Administrators can monitor patient information in real-time through the system, which alerts them if a patient remains in the bathroom for an extended period or ventures beyond a designated safe area, significantly alleviating the burden on nursing staff (https://www.sohu.com/a/www.sohu.com/a/410697273_99976379). However, the application of artificial intelligence technology in ward management also needs to pay attention to ethical issues, such as patient privacy and case data security. Certainly, enhancing medical staff awareness and safety education regarding suicide risk prevention is crucial for safeguarding patients admitted with mental disorders from the peril of suicide (24).

### 4.2 Factors of suicide in hospitalized patients with mental disorders

Our study reveals that psychiatric patients admitted to general hospitals face elevated suicide risk, with multivariate analyses identifying general hospital admission as an independent suicide risk factor. Despite maintaining a psychiatric department, these institutions demonstrate systemic deficiencies in the specialized care protocols. Judicial records from cases like Sun v. Huantai County People’s Hospital [ (2021) Lu 0321 Minchu No. 3618] document critical lapses including inadequate ward segregation, non-standardized patient attire, and deficient monitoring systems. Moreover, mental disorder patients admitted to comprehensive hospitals had a higher suicide rate (n=63, 41.2%) (**Table 1**). Excluding factors intrinsic to individuals with mental disorders, the higher suicide risk among psychiatric patients admitted to general hospitals is primarily due to two interrelated systemic factors: Firstly, general hospitals handle a significantly larger base of initial psychiatric presentations: studies indicate nearly 48% of mental health crises first manifest in non-psychiatric departments of general hospitals. However, these institutions demonstrate suboptimal diagnostic accuracy, with research showing diagnostic error rates as high as 70% (25), where diagnostic error is defined as either delayed, wrong, or missed diagnoses, or failure to effectively communicate accurate diagnoses to patients. Secondly, the management and clinical care protocols for psychiatric inpatients in general hospitals often fail to meet the specialized standards of dedicated psychiatric facilities, particularly regarding multidisciplinary care team integration and diagnostic process optimization—key elements in preventing diagnostic errors according to the National Academy of Medicine framework. To address these systemic challenges, we believe that introducing artificial intelligence-assisted diagnostic technology in general hospital outpatient departments to support physicians in clinical decision-making can effectively alleviate the problem: 1) Deploying AI-assisted diagnostic interfaces in outpatient settings to enhance mental disorder detection among non-specialists; 2) Integrating intelligent monitoring platforms to augment clinical vigilance while reducing staff workload. These interventions aim to bridge the current care gap between physical and mental health services, particularly crucial given that 71% of psychiatric patients globally lack adequate mental healthcare access (https://zhuanlan.zhihu.com/p/666610675).

Additionally, no statistically significant differences were found between hospital levels and regions in the outcome of medical malpractice cases. However, other studies have shown that secondary and tertiary hospitals, along with economically developed provinces, continue to account for a high proportion of psychiatric malpractice cases (26). The pattern may be attributed to the increased awareness of mental health issues in wealthier regions, which leads to higher outpatient visits for mental disorders and higher expectations for facilities that treat mental disorders (27–29). In contrast, disparities in mental health resources across China drive individuals in less developed areas to seek treatment at hospitals with superior medical standards. Combining these factors may explain the large number of psychiatric patients admitted to tertiary hospitals in East and Central China.

Our study suggests that other classes of psychiatric disorders are risk factors for hospitalized patients with mental disorders. This is compared with patients with schizophrenia, schizophrenia, schizotypal, and delusional disorders. This may be related to our classification of mental disorder diagnoses. Mental disorders in other categories include personality disorders, cognitive retardation, organic disorders and other severe mental disorders. Within our study cohort, patients with clinically confirmed schizophrenia accounted for 37 out of 126 (29.4%) suicide-related fatalities, underscoring this diagnosis as a significant predictor of psychiatric inpatient mortality. Clinical studies show that the suicide rate among schizophrenia patients is 4 to 9 times higher than that of the general population (30). During acute episodes, schizophrenia patients often display symptoms such as hallucinations, delusions, and disorganized thinking. This leads to frequent hospitalizations, which increased the risk of suicide-related deaths. Furthermore, the percentage of psychiatric inpatients with recorded suicidal behavior was relatively low in the sample (n=35, 13.1%), but it remains a significant risk factor for mortality. Studies show that a history of suicide attempts is a major predictor of suicide-related fatalities and future attempts (27, 28, 31).

When individuals with mental disorders are admitted to the hospital, an assessment of their suicide risk is essential. Generally, this is determined by the severity of the illness and the need to place the patient on a secure ward. Nevertheless, a review of judicial documents shows that suicide risk assessments are relatively scarce (n=75, 28.0%). Such an assessment may have a significant impact on the medical malpractice liability of healthcare institutions (29). The failure to collect relevant patient data is often the cause of psychiatric errors (32). In cases where clinical personnel determine that a patient is at high risk of suicide, but fail to implement strict safety measures, the institution may be held responsible for medical malpractice. Alternatively, if healthcare staff fail to conduct a suicide risk assessment on admitted patients, the likelihood of self-harm or suicide incidents may increase because of inadequate management. It is important for judges to take into consideration whether evidence of a suicide risk assessment is included in case materials when assessing cases involving psychiatry or medical malpractice. As a result, healthcare institutions should improve their risk assessment protocols for self-harm and suicide, ensuring that each hospitalized patient receives a customized diagnosis and therapeutic intervention.

Inpatient suicide among individuals with mental disorders is significantly influenced by medical negligence during clinical practice. Our analysis categorizes such negligence into three typologies: medical fault in breach of duty to inform, violations of specific regulations, and medical fault in breach of duty of care. Interestingly, these two categories emerged as independent risk factors (OR =3.247, 3.593), indicating that patients exposed to medical regulations violations are 3.247 times more likely to commit suicide than patients treated in compliance, whereas patients receiving substandard care are 3.593 times more likely to commit suicide as compared to those receiving proper care. Generally, this is in accordance with previous studies (33). Our study found that 77.24% of medical institutions were judged to have medical errors in medical dispute cases. This underscores the need to strengthen the supervisory abilities of healthcare professionals and optimize safety management systems within medical facilities, which should be prioritized in order to prevent such tragedies from occurring in the future. There is, however, a shortage of mental health resources in China, which forces psychiatrists and nurses to provide simultaneous treatment to multiple hospitalized patients suffering from mental disorders. Professionals in healthcare have a limited amount of energy. A suicide event may occur if they are not able to provide assistance in a timely manner when patients seek assistance (34). There is evidence that multifaceted, high-quality care reduces the incidence of medical accidents (35). In addition, medical malpractice types can be further classified in a way that provides actionable insights for preventing patient harm. Based on the integration of theoretical frameworks and practical considerations (36), our research team has developed a comprehensive typology of medical harm, providing theoretical and empirical foundations for refining medical malpractice guidelines. Thus, China should implement AI-based warning technologies in hospital ward management to assist healthcare staff in monitoring hospitalized patients and expedite the training of mental health professionals. The use of AI-based monitoring and early warning systems, which rely on massive data analytics, may pose significant risks in the healthcare setting. This type of model is specifically designed to collect video information related to patients from hospital wards continuously, which poses a risk of data breach, potentially compromising sensitive medical records and infringing upon patient rights, which could lead to legal action. In addition, the use of artificial intelligence in these systems raises safety concerns regarding the use of medications: if erroneous nursing data is included in algorithmic decision-making processes, medical staff may commit errors in clinical judgment. The line between human error and technological failure becomes blurred in such scenarios, creating ambiguities in defining nursing liability incidents.

### 4.3 Limitations of the study

The analysis of medical malpractice cases involving hospitalized psychiatric patients contains detailed documentation of suicide characteristics, including temporal patterns, spatial distributions, and methodological approaches. Such granular epidemiological data provide empirical foundations for developing institutional suicide prevention protocols through environmental modifications, staff training on risk recognition, and enhanced monitoring during high-risk periods. By focusing specifically on court-recognized medical faults rather than theoretical frameworks of medical negligence, this approach ensures jurisprudential validity and enhances practical applicability in clinical risk management – particularly given the legal precedent that medical fault determinations must align with contemporaneous professional standards and statutory requirements. However, several limitations should be acknowledged in this research.

In the first place, the study limited its data to psychiatric medical malpractice litigation, which reduces its generalizability. Most incidents, especially those involving suicides, may not result in legal action and are therefore excluded. It may be possible to gain a more accurate and comprehensive picture of the risk factors associated with suicide in inpatient psychiatric settings by incorporating such non-litigated cases into future analyses. Further, the study focuses solely on litigation cases involving suicides of hospitalized individuals with mental disorders. If a case does not proceed to court or liability is not determined, it cannot reflect routine clinical scenarios. Moreover, there may be an attribution bias due to the strong correlation between medical malpractice and suicide events. In order to address these limitations, future research should expand the sample size, establish partnerships with medical institutions, and implement prospective methods such as surveys and structured interviews. By taking these steps, the current findings would be validated and more robust conclusions drawn.

## 5 Conclusion

The study found that there has been a rising trend in suicides among hospitalized patients with mental disorders in China’s medical malpractice cases between 2012 and 2022, with 32.5% of medical malpractice cases involving suicide. Some of the most significant risk factors include treatment in comprehensive hospitals, prior suicidal behavior, clinical diagnoses of severe mental disorders (e.g., schizophrenia), and medical errors, such as regulatory violations and breaches of duty of care. A significant number of suicides occur on weekends, at night, and during the first week of hospitalization, often using tools that are readily available, such as bedsheets. This study emphasizes the need for improved safety protocols, staff training, and the allocation of resources in high-risk settings. The findings highlight systemic gaps in psychiatric care. By dealing with these factors, the mental healthcare system in China may be able to mitigate suicide risks and improve patient outcomes.

## Data Availability

The original contributions presented in the study are included in the article/supplementary material. Further inquiries can be directed to the corresponding author.

## References

[1] Barbosa S Sequeira M Castro S Manso R Câmara CK Trancas B. Causes of death in an acute psychiatric inpatient unit of a portuguese general hospital. Acta Med Port. (2016) 29:468–75. doi: 10.20344/amp.6905, PMID: 27914158

[2] Maddineshat M Khodaveisi M Kamyari N Razavi M Pourmoradi F Sadeghian E. Exploring the safe environment provided by nurses in inpatient psychiatric wards: a mixed-methods study. J Psychiatr Ment Health Nurs. (2024) 31:257–69. doi: 10.1111/jpm.12983, PMID: 37740710

[3] Chammas F Januel D Bouaziz N. Inpatient suicide in psychiatric settings: evaluation of current prevention measures. Front Psychiatry. (2022) 13:997974. doi: 10.3389/fpsyt.2022.997974, PMID: 36386981 PMC9650354

[4] Bhaskaran AS Reddi VSK Suchandra HH Gowda GS Muliyala KP. Predictors of future suicide attempts in individuals with high suicide risk admitted to an acute psychiatry suicide intervention unit in India. A survival Anal study. Asian J Psychiatry. (2022) 78:103270. doi: 10.1016/j.ajp.2022.103270, PMID: 36252324

[5] Malinowska-Lipień I Micek A Gabryś T Kózka M Gajda K Gniadek A. Nurses and physicians attitudes towards factors related to hospitalized patient safety. PloS One. (2021) 16:e0260926. doi: 10.1371/journal.pone.0260926, PMID: 34874957 PMC8651112

[6] De Hert M Cohen D Bobes J Cetkovich-Bakmas M Leucht S M. Ndetei D. Physical illness in patients with severe mental disorders. II. Barriers to care, monitoring and treatment guidelines, plus recommendations at the system and individual level. World Psychiatry. (2011) 10:138–51. doi: 10.1002/j.2051-5545.2011.tb00036.x, PMID: 21633691 PMC3104888

[7] Berg SH Rørtveit K Aase K. Suicidal patients’ experiences regarding their safety during psychiatric in-patient care: a systematic review of qualitative studies. BMC Health Serv Res. (2017) 17:73. doi: 10.1186/s12913-017-2023-8, PMID: 28114936 PMC5259991

[8] Kersting XAK Hirsch S Steinert T. Physical harm and death in the context of coercive measures in psychiatric patients: A systematic review. Front Psychiatry. (2019) 10:400. doi: 10.3389/fpsyt.2019.00400, PMID: 31244695 PMC6580992

[9] Osborn DPJ. The poor physical health of people with mental illness. West J Med. (2001) 175:329–32. doi: 10.1136/ewjm.175.5.329, PMID: 11694483 PMC1071612

[10] Tadmon D Bearman PS. Differential spatial-social accessibility to mental health care and suicide. Proc Natl Acad Sci. (2023) 120:e2301304120. doi: 10.1073/pnas.2301304120, PMID: 37126686 PMC10175830

[11] Goldman-Mellor S Olfson M Lidon-Moyano C Schoenbaum M. Association of suicide and other mortality with emergency department presentation. JAMA Network Open. (2019) 2:e1917571. doi: 10.1001/jamanetworkopen.2019.17571, PMID: 31834399 PMC6991205

[12] Riblet NB Kenneally L Stevens S Watts BV Gui J Forehand J. pilot randomized trial of a brief intervention to prevent suicide in an integrated healthcare setting. Gen Hosp Psychiatry. (2022) 75:68–74. doi: 10.1016/j.genhosppsych.2022.02.002, PMID: 35202942 PMC8955571

[13] Bulut M Mercan N Yüksel Ç. Psikiyatride malpraktis. Psikiyatride Güncel Yaklaşımlar. (2020) 12:195–204. doi: 10.18863/pgy.562489

[14] Jenkins RC D’Alesio DJ Jr. Gann SC. Florida patient safety and pre-suit mediation program for medical malpractice claims: 13-year results, COVID-19 pandemic implications, future innovations, and blueprint for nationwide implementation. J Public Health Issues Pract. (2022) 6(1):193. doi: 10.33790/jphip1100193

[15] Frierson RL. Principles of malpractice litigation in psychiatry. In: Ash P Frierson RL Friedman SH, editors. Malpractice and Liability in Psychiatry. Springer International Publishing, Cham (2022). p. 3–10. doi: 10.1007/978-3-030-91975-7_1

[16] Bartels SJ. The aftermath of suicide on the psychiatric inpatient unit. Gen Hosp Psychiatry. (1987) 9:189–97. doi: 10.1016/0163-8343(87)90007-7, PMID: 3582968

[17] Gale CR Batty GD Osborn DPJ Tynelius P Whitley E Rasmussen F. Mental disorders in early adulthood and later psychiatric hospital admissions in relation to mortality in a cohort study of a million men. Arch Gen Psychiatry. (2012) 69:823–31. doi: 10.1001/archgenpsychiatry.2011.2000, PMID: 22868936 PMC4170756

[18] Mostafapour M Smith JD Fortier JH Garber GE. Beyond medical errors: exploring the interpersonal dynamics in physician-patient relationships linked to medico-legal complaints. BMC Health Serv Res. (2024) 24:1003. doi: 10.1186/s12913-024-11457-3, PMID: 39210366 PMC11361149

[19] Hong M Lee SM Han K-M Kim K-H Paik J-W. Suicide death and other-cause mortality in psychiatric patients: A South Korean study using nationwide claims data. J Affect Disord. (2024) 352:288–95. doi: 10.1016/j.jad.2024.02.075, PMID: 38387668

[20] Cheng ZH Zhang L Wang L Zhang J Kong LJ Yu L. Comparation between guidance for judicial expertise of medical comparation between guidance for judicial expertise of medical. Fa yi xue za zhi. (2022) 38:173–81. doi: 10.12116/j.issn.1004-5619.2022.220205, PMID: 35899501

[21] Luo J Liu H Liu Y Jiang F Tang Y-L. The association between medical liability insurance coverage and medical disturbances in tertiary psychiatric hospitals in China: A national survey. Risk Manage Healthcare Policy. (2021) 14:3767–74. doi: 10.2147/RMHP.S328046, PMID: 34548825 PMC8447944

[22] Tubbs AS Fernandez F-X Perlis ML Hale L Branas CC Barrett M. Suicidal ideation is associated with nighttime wakefulness in a community sample. Sleep. (2021) 44:zsaa128. doi: 10.1093/sleep/zsaa128, PMID: 32614967 PMC8240658

[23] Perlis ML Grandner MA Chakravorty S Bernert RA Brown GK Thase ME. Suicide and sleep: Is it a bad thing to be awake when reason sleeps? Sleep Med Rev. (2016) 29:101–7. doi: 10.1016/j.smrv.2015.10.003, PMID: 26706755 PMC5070474

[24] Mills PD Watts BV Hemphill RR. Suicide attempts and completions on medical-surgical and intensive care units. J Hosp Med. (2014) 9:182–5. doi: 10.1002/jhm.2141, PMID: 24395493

[25] Ibrahim A Hor S Bahar OS Dwomoh D McKay MM Esena RK. Pathways to psychiatric care for mental disorders: a retrospective study of patients seeking mental health services at a public psychiatric facility in Ghana. Int J Ment Health Syst. (2016) 10:63. doi: 10.1186/s13033-016-0095-1, PMID: 27729938 PMC5048657

[26] Pan Y Yang XH He JP Gu YH Zhan XL Gu HF. To be or not to be a doctor, that is the question: a review of serious incidents of violence against doctors in China from 2003–2013. J Public Health. (2015) 23:111–6. doi: 10.1007/s10389-015-0658-7

[27] Nock MK Borges G Bromet EJ Alonso J Angermeyer M Beautrais A. Cross-national prevalence and risk factors for suicidal ideation, plans and attempts. Br J Psychiatry. (2008) 192:98–105. doi: 10.1192/bjp.bp.107.040113, PMID: 18245022 PMC2259024

[28] Klonsky ED May AM Saffer BY. Suicide, suicide attempts, and suicidal ideation. Annu Rev Clin Psychol. (2016) 12:307–30. doi: 10.1146/annurev-clinpsy-021815-093204, PMID: 26772209

[29] Simpson S Stacy M. Avoiding the malpractice snare: documenting suicide risk assessment. J Psychiatr Practice®. (2004) 10:185–9. doi: 10.1097/00131746-200405000-00008, PMID: 15330226

[30] Heilä H Haukka J Suvisaari J Lönnqvist J. Mortality among patients with schizophrenia and reduced psychiatric hospital care. Psychol Med. (2005) 35:725–32. doi: 10.1017/S0033291704004118, PMID: 15918349

[31] Suekane A Takayama W Hashimoto R Morishita K Otomo Y. Risk factors for recurrence of suicide attempt via overdose: A prospective observational study. Am J Emergency Med. (2024) 75:1–6. doi: 10.1016/j.ajem.2023.10.016, PMID: 37890336

[32] Tsao CI Layde JB. A basic review of psychiatric medical malpractice law in the United States. Compr Psychiatry. (2007) 48:309–12. doi: 10.1016/j.comppsych.2007.03.002, PMID: 17560949

[33] Bassett D Tsourtos G. Inpatient suicide in a general hospital psychiatric unit: A consequence of inadequate resources? Gen Hosp Psychiatry. (1993) 15:301–6. doi: 10.1016/0163-8343(93)90022-G, PMID: 8307343

[34] Firth J Siddiqi N Koyanagi A Siskind D Rosenbaum S Galletly C. The Lancet Psychiatry Commission: a blueprint for protecting physical health in people with mental illness. Lancet Psychiatry. (2019) 6:675–712. doi: 10.1016/S2215-0366(19)30132-4, PMID: 31324560

[35] Alexander DA. Suicide by patients: questionnaire study of its effect on consultant psychiatrists. BMJ. (2000) 320:1571–4. doi: 10.1136/bmj.320.7249.1571, PMID: 10845964 PMC27400

[36] Wang Q Sun D-M. Applicability dilemmas and practical approaches for judicial expertise standards in psychiatric medical malpractice. Chin J Forensic Sci. (2024) 1:76–83. doi: 10.3969/j.issn.1671-2072.2024.03.011

